# Oxidative Stress and Inflammation in SARS-CoV-2- and *Chlamydia pneumoniae*-Associated Cardiovascular Diseases

**DOI:** 10.3390/biomedicines9070723

**Published:** 2021-06-24

**Authors:** Simone Filardo, Marisa Di Pietro, Fabiana Diaco, Silvio Romano, Rosa Sessa

**Affiliations:** 1 Department of Public Health and Infectious Diseases, University of Rome “Sapienza”, P.le Aldo Moro, 5, 00185 Rome, Italy; marisa.dipietro@uniroma1.it (M.D.P.); fabiana.diaco@uniroma1.it (F.D.); rosa.sessa@uniroma1.it (R.S.); 2 Cardiology, Department of Life, Health and Environmental Sciences, University of L’Aquila, P.le Salvatore Tommasi, 1, 67100 L’Aquila, Italy; silvio.romano@univaq.it

**Keywords:** SARS-CoV-2, *Chlamydia pneumoniae*, oxidative stress, inflammation, antioxidants, cardiovascular diseases

## Abstract

Throughout the years, a growing number of studies have provided evidence that oxidative stress and inflammation may be involved in the pathogenesis of infectious agent-related cardiovascular diseases. Amongst the numerous respiratory pathogens, severe acute respiratory syndrome coronavirus 2 (SARS-CoV-2), a novel coronavirus responsible for the global ongoing pandemic, and *Chlamydia pneumoniae*, a widely known intracellular obligate bacteria, seem to have an essential role in promoting reactive oxygen species and cytokine production. The present review highlights the common oxidative and inflammatory molecular pathways underlying the cardiovascular diseases associated with SARS-CoV-2 or *C. pneumoniae* infections. The main therapeutic and preventive approaches using natural antioxidant compounds will be also discussed.

## 1. Introduction

Over the past decades, oxidative stress and inflammation have been identified as relevant pathophysiological pathways in the development of cardiovascular diseases (CVDs), with increasing evidence showing their complex interplay in all the stages leading to CVDs, from endothelial dysfunction to thrombosis [1,2].

Oxidative stress is defined as the imbalance between the production of reactive oxygen species (ROS) and the endogenous antioxidant defense systems, termed the redox state [3]. ROS, including free oxygen radicals, oxygen ions, and peroxides, act as signaling molecules under physiological conditions for the defense against invading microorganisms and are essential in cell growth and proliferation and inflammatory responses [3]. When the release of ROS is not limited by antioxidant defense systems, oxidative stress causes cellular dysfunction, protein and lipid peroxidation, and DNA damage, leading to irreversible cell damage [3].

In the cardiovascular system, ROS are produced by several enzyme systems, including nicotinamide adenine dinucleotide phosphate (NAPDH) oxidase (NOX), xanthine oxidase (XO), uncoupled endothelial nitric oxide synthase (eNOS), and the mitochondrial electron transport chain [4,5]. On the other hand, the vasculature and cardiomyocytes are protected by antioxidant enzyme systems, including superoxide dismutase (SOD), catalase, glutathione peroxidases (GPx), and paraoxonases, which detoxify ROS [4].

Additionally, inflammation, known as a primary protective response to tissue damage or infection, is a complex process that occurs in the vascular tissue involving inflammatory immune cells, interactions between cell surfaces, and proinflammatory mediators [6,7]. The link between oxidative stress and inflammation has been demonstrated from an increased production of adhesion molecules, resulting in the migration and infiltration of inflammatory cells in the vascular tissue, following low-density lipoprotein (LDL) oxidation [8]. Activated monocytes, lymphocytes, and mast cells, in turn, produce ROS, chemokines, interleukins, and proteases, worsening the inflammatory state with detrimental effects on vascular tissue [7,8].

Throughout the years, a growing number of studies have provided evidence that diabetes, dyslipidemia, and obesity are the major risk factors involved in the pathogenesis of CVDs by enhancing oxidative stress as well as inflammation [9,10,11]. More recently, respiratory pathogens have also been shown to alter the host redox balance and elicit a damaging inflammatory response, contributing to cardiovascular complications. Amongst them, severe acute respiratory syndrome coronavirus 2 (SARS-CoV-2), a novel coronavirus, and *Chlamydia pneumoniae*, a widely known intracellular obligate bacteria, seem to have an essential role in promoting ROS and cytokine production.

The present review highlights the common oxidative and inflammatory molecular pathways underlying the cardiovascular diseases associated with SARS-CoV-2 or *C. pneumoniae* infections. The main therapeutic and preventive approaches using natural antioxidant compounds will be also discussed.

## 2. General Characteristics of SARS-CoV-2 and *C. pneumoniae*

SARS-CoV-2, a novel respiratory virus that first emerged in Wuhan, China, in December 2019, is the causative agent of a severe acute respiratory syndrome, responsible for a pandemic declared as a global health emergency by the World Health Organization (WHO) at the end of January 2020. This pathogen is a new type of coronavirus whose origin is still unclear, although genome sequencing showed a homology of more than 79% with SARS-CoV, hence the denomination of SARS-CoV-2 [12]. However, the newly discovered human coronavirus is characterized by a 10–20% higher infectivity and transmissibility and higher lethality than SARS-CoV, with more than 168 million confirmed cases, including 3.49 million deaths, as of 28 May 2021 (https://covid19.who.int/ (accessed on 28 May 2021)).

The clinical presentation of SARS-CoV-2 infection, defined as Coronavirus Disease 2019 (COVID-19), ranges from “flu-like” symptoms, including fever, headache, shortness of breath, and myalgia, to, in some cases, severe pneumonia with respiratory failure (acute respiratory distress syndrome, ARDS) and, ultimately, a fatal outcome [12]. However, a significant proportion of Sars-CoV-2 cases are asymptomatic, favoring the transmission in the population, although the size and characteristics of the asymptomatic subpopulation remain poorly understood, with studies reporting estimates of asymptomatic Sars-CoV-2 infections from as low as 4% to more than 80% [13].

Although SARS-CoV-2 primarily targets the lungs, some patients have developed clinical manifestations in other organs and systems, including the heart and blood vessels. Indeed, acute cardiac injury with elevated troponin levels was reported in approximately 8–12% of all SARS-CoV-2-positive patients, with 33% of critically ill patients developing a cardiomyopathy [14,15,16,17]. Moreover, a systematic review of cardiac autopsies in COVID-19 patients reported a high detection rate of viral RNA in the myocardium with frequent nonmyocardial infarction pattern fibrosis, consistent with microvascular ischemia/thrombi and, in some cases, endothelial inflammation [18]. Indeed, it has been recently demonstrated that SARS-CoV-2 is able to infect the endothelium, leading to endothelial dysfunction that can result in predisposition to thrombosis in all arterial beds of the microvasculature, including the pulmonary and coronary circulation as well as the peripheral veins and arteries of the cerebral circulation, potentially causing strokes [19,20]. This is further confirmed by the high D-dimer levels found in 20–40% of critically ill patients as an attempt to dissolve thrombotic clots [21].

The SARS-CoV-2 infection of a broad range of different tissues in the host is explained by the expression of high levels of Angiotensin-converting enzyme (ACE)-2 receptors on the cell surface [22]. In this regard, ACE2 has recently acquired importance in the pathogenesis of SARS-CoV-2 infection for its role as a functional point of entry for the virus by binding to its surface S protein [22] as well as for its important regulatory role in the renin-angiotensin-aldosterone system (RAAS) [23]. ACE2 is also expressed in the heart and the vascular endothelium, and SARS-CoV-2 has been shown to bind with high affinity to these receptors [22,23], invading and replicating within myocardial and endothelial cells as well as pericytes [24,25], leading to tissue damage.

*C. pneumoniae* is known as the etiologic agent of respiratory tract infections in humans, such as community-acquired pneumonia, bronchitis, pharyngitis, and sinusitis [26]. Pneumonia, responsible for 10–20% of cases, can rarely lead to respiratory failure and death [26]. Nevertheless, most infections are asymptomatic (70%) or manifest with mild to moderate symptoms [26].

Exposure to *C. pneumoniae* is extremely common as evidenced from the high prevalence of antibodies in the general population; indeed, more than half of the world population is seropositive to *C. pneumoniae* [26]. Again, *C. pneumoniae* infection could be acquired early in life and persist over time as suggested by epidemiological studies showing a 50% antibody prevalence by the age of 20 and 80% by the age of 60 to 70 [26].

*C. pneumoniae* is an intracellular obligate pathogen with a unique developmental cycle, characterized by two alternating functionally and morphologically distinct forms: the elementary body, the metabolically inert and infectious form, and the reticulate body, the intracellular replicative form [27].

In some conditions, such as treatment with certain antibiotics, the exposure to Interferon (IFN)-γ, and specific cells, such as monocytes/macrophages, *C. pneumoniae* fails to complete its developmental cycle, generating persistent forms which remain viable but noninfectious inside the host cell for a long time due to their ability to evade the immune system [27].

A peculiar feature of *C. pneumoniae* is the ability to systematically disseminate from the lungs through peripheral blood mononuclear cells (PBMCs) and to localize in several extrapulmonary tissues, including the vasculature [28,29,30,31,32,33,34,35,36,37]. Indeed, *C. pneumoniae* has long been associated with several chronic inflammatory diseases with a great impact on public health, mainly atherosclerosis [35,38,39,40,41,42,43]. Other pathogens have been associated with atherosclerosis, such as, for example, periodontal bacteria and Helicobacter pylori, although *C. pneumoniae* is considered as the most implicated infectious agent in the pathogenesis of atherosclerotic CVDs by extensive evidence, including seroepidemological data and the direct detection of this pathogen in atherosclerotic plaque [44]. This has been further supported by in vivo studies demonstrating that *C. pneumoniae* infection may accelerate the progression of atherosclerotic lesion in animal models and in vitro studies showing that *C. pneumoniae* is able to multiply in all cell types involved in the pathogenesis of atherosclerosis, including monocytes/macrophages, vascular endothelial, and smooth muscle cells (VSMCs) [33,44,45,46,47].

## 3. Cellular and Molecular Pathways Related to Oxidative Stress and Inflammation in SARS-CoV-2 and *C. pneumoniae* Infections

### 3.1. SARS-CoV-2

The first evidence that oxidative stress might play a role in COVID-19 infection was provided by clinical studies investigating the oxidants–antioxidants balance in patients with moderate to severe forms of the disease [48,49,50,51,52]. In this regard, a cross-sectional study showed reduced levels of antioxidant vitamins (vitamin A, C, and E), enzymes (glutathione, superoxide dismutase, and catalase), and trace elements (manganese, zinc, selenium, etc.) in COVID-19 patients, suggesting an altered host redox state [49]. More importantly, the downregulation of redox-active genes, such as superoxide dismutase 3 (SOD3), activating transcription factor 4 (ATF4), and metallothionein 2A (M2TA), observed in the lungs of elderly COVID-19 patients seemed to be connected to the severity of the disease [50]. A stronger confirmation came from studies demonstrating that decreased levels of antioxidants were accompanied by increased oxidative stress, as evidenced by lipid peroxidation as well as higher levels of reactive oxygen and nitrogen species in patients with severe SARS-CoV-2 [51,52].

In fact, it is known that the progressive failure of major antioxidant defense mechanisms to respond to ROS-induced damage occurs physiologically due to aging, and this may explain the increased severity of COVID-19 symptoms in older people, alongside the higher incidence of cardiovascular complications [53,54]. In this regard, organs such as the heart are particularly vulnerable to oxidative stress for their high rates of oxygen consumption, hence contributing to the high prevalence of CVDs in the elderly [54].

**Figure 1:**
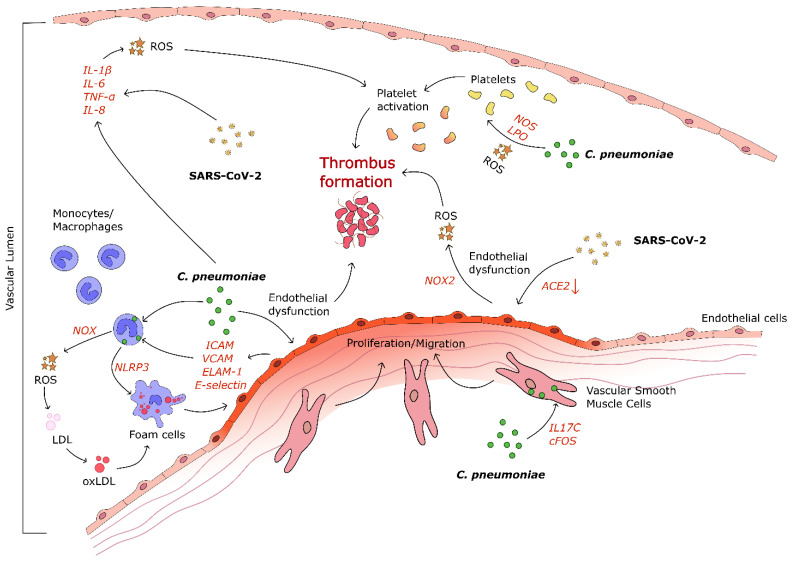
Cellular and molecular pathways involved in SARS-CoV-2- and *C. pneumoniae* -mediated vascular diseases. SARS-CoV-2 contributes to increased inflammation, endothelial dysfunction, and, ultimately, thrombus formation. *C. pneumoniae* induces inflammatory cytokine production, endothelial dysfunction, foam cell formation, vascular smooth muscle cell (VSMC) migration, and proliferation to intima, leading to thrombus formation. ACE-2, angiotensin converting enzyme-2; ROS, reactive oxygen species; NOX-2, nicotinamide adenine dinucleotide phosphate (NAPDH) oxidase-2; IL, interleukin; TNFα, tumor necrosis factor; NOS, nitric oxide synthase; LOS, lipoxygenase; LDL, low-density lipoprotein; NLRP-3, nod-like receptor family pyrin domain-containing 3; ICAM, intercellular adhesion molecule; VCAM, vascular cell adhesion molecule; ELAM-1, endothelial-leukocyte adhesion molecule-1. The red arrow indicates decrease in marker’s levels.

All the clinical evidence led to the postulation that numerous mechanisms (Figure 1) might explain the link between SARS-CoV-2 infection and increased oxidative stress contributing to the development of extrapulmonary complications, such as CVDs, related to the more severe forms of COVID-19 [55].

Specifically, SARS-CoV-2 has been demonstrated to infect endothelial cells and, hence, induce endothelial dysfunction and vascular inflammation via the downregulation of ACE2 expression on the target cell surface, causing the imbalance of the renin-angiotensin-aldosterone (RAAS) system and triggering the production of reactive oxygen species (ROS) via NOX activation and reduced availability of nitric oxide (NO) via decreased eNOS activity [20,56]. Indeed, a higher NOX-2 activation was observed in patients with thrombotic complications as compared to event-free patients, suggesting that NOX-2-derived oxidative stress contributed to the pathophysiology of COVID-19 cardiovascular sequelae [57]. As an additional mechanism, SARS-CoV-2 infection might lead to oxidative stress and alter mitochondrial function through the dysregulation of several genes related to protein SUMOylation, the regulation of glucocorticoid biosynthesis, and cellular response to stress [58].

Concerning inflammation, SARS-CoV-2 infection strongly activates innate immune pathways, triggering an uncontrolled cytokine response named “cytokine storm” that targets several tissue and organs, including the endothelial cells that, in turn, release proinflammatory cytokines and chemokines that recruit immune cells into the site of inflammation [59,60]. These are believed to play an important role in the hyperinflammation that characterizes patients with severe forms of COVID-19, releasing large amount of proinflammatory cytokines (for example, interleukin IL-1β, IL-6, tumoral necrosis factor TNF-α, and IL-8) that might promote free radical production and oxidative stress [48]. This is, indeed, strongly suggested by evidence that other respiratory viral pathogens, such as influenza virus, human respiratory syncytial virus, rhinovirus, and SARS-CoV-1, have been shown to elicit excessive amount of ROS production through different mechanisms, including the strong inflammatory activation of immune cells [48]. Specifically, nonstructural viral proteins of SARS-CoV-1, such as the coronavirus 3a protein, have been demonstrated to activate the nod-like receptor family pyrin domain-containing (NLRP)-3 inflammasome in macrophages, leading to IL-1β production and increased mtROS levels [61]. Hence, it is highly likely that similar mechanisms may also be employed by SARS-CoV-2.

### 3.2. Chlamydia pneumoniae

In the past 30 years, different lines of evidence have supported the involvement of *C. pneumoniae* in the pathogenesis of atherosclerosis, the underlying pathological process of CVDs [62].

Particularly important are the molecular studies that have highlighted oxidative stress and inflammation as the most likely pathogenic mechanisms by which *C. pneumoniae* may contribute to the early as well as late stages of the atherosclerotic process by promoting endothelial dysfunction, foam cell formation, platelet activation, and thrombus formation (Figure 1) [63].

As for endothelial dysfunction, characterized by increased production of anion superoxide and reduced NO bioavailability, *C. pneumoniae* has been shown to interfere with multiple enzymatic systems involved in ROS production and detoxification [64]. Specifically, *C. pneumoniae* has been demonstrated to elicit ROS overproduction by upregulating NOX and cyclooxygenase (COX-2) and downregulating antioxidant enzyme systems, such as catalase, SOD-1, and thioredoxin-1 [65]. There is also evidence that *C. pneumoniae*-induced oxidative stress may contribute to endothelial dysfunction by decreasing eNOS expression and, hence, NO synthesis in endothelial cells [66,67].

Notably, the ability of *C. pneumoniae* to modulate the expression of enzymes related to ROS production and detoxification has also been observed in monocytes/macrophages [64]. Indeed, *C. pneumoniae* stimulates superoxide anion production via the NOX pathway and, at the same time, increases the antioxidant activity of cytochrome c oxidase and other antioxidant enzyme systems, such as SOD, GPx, and γ-glutamylcysteine synthase (γ-GCS), paradoxically attenuating ROS release [68]. As a result, *C. pneumoniae* is able to survive in monocytes/macrophages, considered as a reservoir of chronic infection; to stimulate LDL oxidation and foam cell formation; and to augment cell necrosis, leading to plaque progression.

*C. pneumoniae*-mediated oxidative stress has also been shown to regulate the functions of platelets and vascular smooth muscle cells (VSMCs) [69]. In platelets, *C. pneumoniae*-induced ROS production via the nitric oxide synthase (NOS) and LOX pathways has been described to mostly contribute to their activation and aggregation and, consequently, to thrombotic vascular occlusion [70]. In VSMCs, *C. pneumoniae* has been demonstrated to elicit ROS production in the extracellular compartment that may inactivate the vasoprotective molecule NO and, thus, contribute to endothelial dysfunction [71].

In addition to oxidative stress, *C. pneumoniae* is known to induce a chronic inflammatory response via the mitogen-activated protein kinase and nuclear factor-κB pathways, further exacerbating the atherosclerotic process [35]. Indeed, cytokines (IL-6, IL-8, and TNF-α), chemokines (monocytes chemoattract protein, MCP-1), and adhesion molecules (endothelial-leukocyte adhesion molecule, ELAM-1; intercellular adhesion molecule, ICAM-1; and vascular cell adhesion molecule, VCAM-1) produced by vascular cells after exposure to *C. pneumoniae* have been reported to increase the migration of leukocytes and VSMCs to the vascular wall, thus contributing to plaque destabilization [69,71,72].

More recently, the crosstalk between IL-17C and c-Fos, a component of activator protein 1 (AP-1), has been described as a new regulatory mechanism activated by *C. pneumoniae* and responsible for VSMC migration to the intima [73]. In addition to vascular inflammation, *C. pneumoniae* has also been shown to contribute to the systemic inflammation involved in the pathogenesis of atherosclerotic cardiovascular diseases [74].

Lastly, a link between oxidative stress and inflammation has been provided by compelling evidence for the role of *C. pneumoniae*-induced ROS production, alongside dyslipidemia, in the activation of the nod-like receptor family pyrin domain-containing (NLRP)-3 inflammasome, with a subsequent increase of IL-1β and accumulation of intracellular cholesterol and foam cell formation [75,76].

## 4. Antioxidant Strategies against SARS-CoV-2 and *C. pneumoniae*

It is of utmost importance to address the imbalance of host redox stress to mitigate the infection-mediated tissue damage leading to the development of cardiovascular complications following SARS-CoV-2 and *C. pneumoniae* infections.

The available evidence to date on the efficacy of natural compounds, including high-dose zinc and ascorbic acid or inhaled nitric oxide, targeting SARS-CoV-2-mediated oxidative stress is controversial. Indeed, a clinical trial observed a decreased severity and reduced lethal outcomes of COVID-19 infection after treatment with antioxidant supplements, such as vitamins C and E, N-acetylcysteine, melatonin, and pentoxifylline [77]. Moreover, a prospective study investigating the effect of inhaled nitric oxide administration in COVID-19 patients with severe pneumonia showed an improvement of pulmonary circulation in the majority of patients [78]. However, more randomized clinical trials reported no significantly reduced symptom duration, days of hospitalization, proportion of patients requiring intubation, or overall mortality after antioxidant supplementation (high-dose zinc and ascorbic acid and inhaled nitric oxide) in COVID-19 patients with severe manifestations [79,80,81,82].

It is worth noting that the controversial outcomes in inhaled NO trials might be attributed to differences in treatment time and NO concentrations [83]. In fact, it is well known that NO expresses a broad spectrum of concentration-dependent biological effects, ranging from antiviral activity and vasodilation at low doses, favoring oxygenation and tissue perfusion, to harmful effects at high concentrations, eventually leading to cell death and tissue damage [84,85].

Nevertheless, data on the antioxidant treatment against SARS-CoV-2-mediated oxidative stress are limited at the time of writing due to the fact that most randomized clinical trial are still at the early stages, investigating vitamin C and melatonin [86,87,88,89,90,91,92]. However, there are a plethora of other potential supplements, such as, for example, resveratrol, probiotic/synbiotic, magnesium, and natural plant extracts, that have been demonstrated to decrease oxidative stress and inflammation, although there are no data on their effects toward SARS-CoV-2 [93,94,95,96].

Concerning the antioxidant treatment against *C. pneumoniae*, several natural compounds well known for their beneficial health properties, such as curcumin (1 µM), resveratrol (25 µM), and vitamin E (50 µM), have been suggested over the course of several years as intriguing candidates due to their in vitro efficacy in reducing ROS production [69,97,98]. Other natural compounds, such as lignans (25–100 µM) from *Schisandra chinensis* berries, known for their antioxidative and cytoprotective properties, have been shown to reduce ROS intracellular levels and to inhibit *C. pneumoniae* growth [99].

Another antioxidant strategy may be represented by substances able to mimic the biochemical activity of ROS detoxifying enzymes. For example, Mn (III) tetrakis (4-benzoic acid) porphyrin chloride (MnTBAP) was demonstrated to stimulate NOS activity in endothelial cells [66], and sesamol (10 µg/mL), the main component of sesame seed oil, was shown to inhibit *C. pneumoniae*-mediated VSMC proliferation [100].

In addition to the encouraging effects of antioxidants in in vitro studies, a meta-analysis of randomized clinical trials has shown that there is no evidence to support the use of vitamins for the prevention of CVDs [101], although a recent clinical trial has shown that lycopene, a member of the carotenoid family with antioxidant properties, decreased the levels of oxidized LDL and tissue damage, as well as the levels of *C. pneumoniae* IgG, in patients with coronary vascular disease [102].

## 5. Conclusions and Future Perspectives

Despite the different natures of SARS-CoV-2 and *C. pneumoniae*, the first a novel respiratory virus and the latter an intracellular obligate bacterium, both depend on the host cell for their replication and possess high tropism for lung tissue, the primary site of infection and starting point for the dissemination for either of these pathogens in the host organism. SARS-CoV-2 and *C. pneumoniae* localize in a broad range of tissues and organs, such as the heart and vasculature, likely leading to tissue damage and hence contributing to cardiovascular complications. In fact, SARS-CoV-2 and *C. pneumoniae* share some cellular and molecular pathways in endothelial dysfunction, thrombus formation, and ROS and proinflammatory cytokine production.

Further common clinical features include the high prevalence of asymptomatic infections and the ability to induce long term damage in the host organism. Indeed, a post-COVID-19 syndrome, characterized by single- or multiorgan impairment, involving, for example, the heart, has been described to persist after SARS-COV-2 viral clearance [103,104,105]. Similarly, *C. pneumoniae* is known to persist in vascular cells, contributing to the typical changes of atherosclerotic plaque, as evidenced by the presence of chlamydial DNA in PBMCs as well as in atherosclerotic lesions [32,106,107,108].

Lastly, as for the usage of antioxidant natural compounds, several approaches have been attempted for SARS-CoV-2 and *C. pneumoniae* infections, although with controversial outcomes, especially in clinical trials.

Particularly interesting are the recent studies evidencing SARS-CoV-2/*C. pneumoniae* coinfection in COVID-19 patients [109,110]. In this regard, De Francesco et al. (2021) found an association between the presence of SARS-CoV-2/*C. pneumoniae* coinfection and the severity of the COVID-19 disease [110]. Such observations may indeed open a new pathophysiological scenario; *C. pneumoniae*, acquired early in life, may contribute to the cytokine storm observed in severe COVID-19 disease for its ability to generate persistent forms believed to be responsible for local and systemic chronic inflammation [60]. Additionally, the oxidative mechanisms related to *C. pneumoniae* may be involved in severe COVID-19 disease, since the ROS production in platelets following chlamydial infection has been described to contribute to thrombus formation.

In conclusion, a significant amount of evidence suggests that both SARS-CoV-2 and *C. pneumoniae* infections may be involved in the development of cardiovascular diseases through oxidative stress and inflammation, although many questions still remain unanswered, such as, for example, the role of coinfection in long-term damage.

In the future, increased knowledge on SARS-CoV-2- and *C. pneumoniae*-mediated vascular damage, alongside the identification of novel antioxidant strategies, will be of great help to complete the whole pathophysiologic picture.
